# Supplementary material for: A Global Survey of Ethnic Indian Women Living with Polycystic Ovary Syndrome: Co-Morbidities, Concerns, Diagnosis Experiences, Quality of Life, and Use of Treatment Methods
Source: Int J Environ Res Public Health. 2022 Nov 28;19(23):15850. doi: 10.3390/ijerph192315850 (PMC9740300; doi:10.3390/ijerph192315850)
Supplement: Supplementary file 1 [file ijerph-19-15850-s001.zip › ijerph-1943089-supplementary.pdf]

Polycystic Ovary Syndrome (PCOS) in ethnic Indian women: a global survey

1. **Before we begin, let's make sure that you are eligible for this survey.**

   Do you identify yourself as a woman of Indian ethnic ancestry? (Have at least one parent or grandparent who was born in India)

- ○ Yes (1)
- ○ No (2)

Q3 Have you been diagnosed with PCOS by a medical doctor?

- ○ Yes (1)
- ○ No (2)

Q4
What is your age in years?

▼ 18 (1) ... 55 (38)

Q5
What is your current body weight in kilograms? (use this convertor if needed to convert pounds to kilograms)

____

Q6 What is your height in centimetres? (use this convertor if needed to convert feet and inches to centimetres)

____

Q7 Which of the following health conditions do you have other than PCOS? (multiple answers)

- ▢ Anxiety (1)
- ▢ Chronic fatigue (2)
- ▢ Coronary artery disease (myocardial infarction/heart attacks and/or angina) (3)
- ▢ Depression (4)
- ▢ Eating disorder (5)
- ▢ Endometrial cancer (6)
- ▢ High blood pressure (7)
- ▢ High cholesterol levels or triglyceride levels (8)
- ▢ Sleep disorders (eg. sleep apnea) (9)
- ▢ Thyroid related problems (eg. hypothyroid, hyperthyroid) (10)
- ▢ Type 2 diabetes (11)
- ▢ ⊗I don't have any other health condition other than PCOS (12)
- ▢ Other than above (please specify) (13)

____

Q8 Which of the following immediate family members have been diagnosed with PCOS? (multiple answers)

- ▢ Mother (1)
- ▢ Sister (2)
- ▢ Daughter (3)
- ▢ ⊗None of the above (4)

Q9 Which of the following immediate family members have been diagnosed with type 2 diabetes? (multiple answers)

- ▢ Brother (1)
- ▢ Daughter (2)
- ▢ Father (3)
- ▢ Mother (4)
- ▢ Sister (5)
- ▢ Son (6)
- ▢ ⊗None of the above (7)

Q10 **Now, please share your experience of diagnosis and signs and symptoms of PCOS.** How old were you when you first experienced the signs and symptoms of PCOS?

▼ Less than 10 (1) ... 55 (47)

Q11 How old were you when you first visited a doctor for any of the above signs and symptoms?

▼ Less than 10 (1) ... 55 (47)

Q12 At what age were you diagnosed with PCOS?

▼ Less than 10 (1) ... 55 (47)

Q13 Which country you were living in when you were diagnosed with PCOS?

▼ Afghanistan (1) ... Zimbabwe (195)

Q14 Who performed the diagnosis?

- ○ Cardiologist (1)
- ○ Dermatologist (2)
- ○ Endocrinologist (3)
- ○ Gynecologist/obstetrician (4)
- ○ General practitioner/family physician/family doctor (5)
- ○ Infertility specialist (6)
- ○ Psychiatrist (7)

Q15 Which of the following signs and symptoms did you first experience? (multiple answers)

- ▢ Acne/pimples (1)
- ▢ Anxiety (2)
- ▢ Cysts on the ovaries (in an ultrasound) (3)
- ▢ Depression (4)
- ▢ Difficulty losing weight (5)
- ▢ Excess unwanted hair growth over face (6)
- ▢ Excess hair loss (7)
- ▢ High blood levels of androgens/male hormones (eg. testosterone) (8)
- ▢ Increased metabolic risk (eg. fear of developing type 2 diabetes) (9)
- ▢ Increased tendency for weight gain (10)
- ▢ Irregular menstrual cycles/periods (11)
- ▢ Problems with ovulation (12)
- ▢ Not able to fall pregnant (Infertility) (13)
- ▢ ⊗I don't remember (14)
- ▢ Other than above (please specify) (15)

____

Q16 How satisfied were you with the manner in which you were informed of the diagnosis?

- ○ Very dissatisfied (1)
- ○ Dissatisfied (2)
- ○ Neither satisfied nor dissatisfied (3)
- ○ Satisfied (4)
- ○ Very satisfied (5)

Q17 How satisfied were you with the information given about : (select below)

	Information was not given (1)	Very dissatisfied (2)	Dissatisfied (3)	Neither satisfied now dissatisfied (4)	Satisfied (5)	Very satisfied (6)
PCOS (1)	▢	▢	▢	▢	▢	▢
Long term complications (2)	▢	▢	▢	▢	▢	▢
Treatment options (3)	▢	▢	▢	▢	▢	▢
Appropriate dietary advice (4)	▢	▢	▢	▢	▢	▢
Appropriate exercise advice (5)	▢	▢	▢	▢	▢	▢
Appropriate behavioral advice to improve diet or exercise (6)	▢	▢	▢	▢	▢	▢
Emotional support after diagnosis (7)	▢	▢	▢	▢	▢	▢

Q18 Which of the following signs and symptoms of PCOS concern you the most? **(Please select any three)**

- ▢ Acne/pimples (1)
- ▢ Anxiety (2)
- ▢ Cysts on the ovaries (in an ultrasound) (3)
- ▢ Depression (4)
- ▢ Difficulty losing weight (5)
- ▢ Excess unwanted hair growth over face (6)
- ▢ Excess hair loss (7)
- ▢ High blood levels of androgens/male hormones (eg. testosterone) (8)
- ▢ Increased metabolic risk (eg. fear of developing type 2 diabetes) (9)
- ▢ Increased tendency for weight gain (10)
- ▢ Irregular menstrual cycles/periods (11)
- ▢ Problems with ovulation (12)
- ▢ Not able to fall pregnant (Infertility) (13)
- ▢ Other than above (please specify) (14)
  ____

Q19 Duirng the last two weeks, to what extent the followings issues have been a problem for you:

	A severe problem (1)	A major problem (2)	A moderate problem (3)	Some problem (4)	A little problem (5)	Hardly any problem (6)	No problem (7)
Growth of visible body hair (7)	○	○	○	○	○	○	○
Growth of visible hair on your face? (2)	○	○	○	○	○	○	○
Growth of visible hair on chin? (3)	○	○	○	○	○	○	○
Growth of visible hair on upper lip? (4)	○	○	○	○	○	○	○
Acne (6)	○	○	○	○	○	○	○

Q20 During the past two weeks, how much of the time have you felt:

Display This Choice:

If Duirng the last two weeks, to what extent the followings issues have been a problem for you: != Acne [No problem]

Display This Choice:

If Duirng the last two weeks, to what extent the followings issues have been a problem for you: != Acne [No problem]

Display This Choice:

If Duirng the last two weeks, to what extent the followings issues have been a problem for you: != Growth of visible body hair [No problem]

	All of the time (1)	Most of the time (2)	A good bit of the time (3)	Some of the time (4)	A little of the time (5)	Hardly any of the time (6)	None of the time (7)
Depressed as a result of having PCOS? (1)	○	○	○	○	○	○	○
Concerned about being overweight? (2)	○	○	○	○	○	○	○
Easily tired? (3)	○	○	○	○	○	○	○
Concerned about infertility problems? (4)	○	○	○	○	○	○	○
Moody as a result of having PCOS? (5)	○	○	○	○	○	○	○
Display This Choice: If Duirng the last two weeks, to what extent the followings issues have been a problem for you: != Acne [No problem] Unattractive because of acne? (6)	○	○	○	○	○	○	○

Display This Choice: *If Duirng the last two weeks, to what extent the followings issues have been a problem for you: != Acne [No problem]* Depressed because of acne? (7)	○	○	○	○	○	○	○
Display This Choice: *If Duirng the last two weeks, to what extent the followings issues have been a problem for you: != Growth of visible body hair [No problem]* Embarrassment about excessive body hair? (8)	○	○	○	○	○	○	○

Q21 During the past two weeks, how much of the time have you:

	All of the time (1)	Most of the time (2)	A good bit of the time (3)	Some of the time (4)	A little of the time (5)	Hardly any of the time (6)	None of the time (7)
Had trouble dealing with your weight? (1)	○	○	○	○	○	○	○
Had low self-esteem as a result of having PCOS? (2)	○	○	○	○	○	○	○
Felt frustration in trying to lose weight? (3)	○	○	○	○	○	○	○
Felt afraid of not being able to have children? (4)	○	○	○	○	○	○	○
Felt frightened of getting cancer? (5)	○	○	○	○	○	○	○
Been worried about having PCOS? (6)	○	○	○	○	○	○	○
Been self-conscious as a	○	○	○	○	○	○	○

result of having PCOS? (7)	

Q22 How much of the time during the last two weeks did you:

	All of the time (1)	Most of the time (2)	A good bit of the time (3)	Some of the time (4)	A little of the time (5)	Hardly any of the time (6)	None of the time (7)
Feel like you are not sexy because of being overweight? (1)	○	○	○	○	○	○	○
Feel lack of control over the situation with PCOS? (2)	○	○	○	○	○	○	○
Have difficulties staying at your ideal weight? (3)	○	○	○	○	○	○	○
Feel sad because of infertility problems? (4)	○	○	○	○	○	○	○

Q23 In relation to your last menstruation, how much were the following issues a problem for you:

	A severe problem (1)	A major problem (2)	A moderate problem (3)	Some problem (4)	A little problem (5)	Hardly any problem (6)	No problem (7)
Headaches (1)	○	○	○	○	○	○	○
Irregular cycles (2)	○	○	○	○	○	○	○
Abdominal bloating (3)	○	○	○	○	○	○	○
Late menstrual periods (4)	○	○	○	○	○	○	○
Menstrual cramps (5)	○	○	○	○	○	○	○
Acne (6)	○	○	○	○	○	○	○

Q24 So far, which of the following health practitioners have you consulted for PCOS? (multiple answers)

- ▢ Allied health professionals (eg. dietician, exercise physiologist) (1)
- ▢ Cardiologist (2)
- ▢ Dermatologist (3)
- ▢ Endocrinologist (4)
- ▢ General practitioner/family physician/family doctor (5)
- ▢ Gynecologist/obstetrician (6)
- ▢ Infertility specialist (7)
- ▢ Psychiatrist (8)
- ▢ ⊗Never seen a medical doctor or allied health practitioner for PCOS treatment (9)
- ▢ Other than above (please specify) (10) ____

Skip To: End of Survey If So far, which of the following health practitioners have you consulted for PCOS? (multiple answers) =

Q25 Which of the following medical treatment methods have you used to manage PCOS? (multiple answers)

- ▢ Anti-androgen drugs (to correct male-hormone levels) (1)
- ▢ Anti-obesity drugs (2)
- ▢ Bariatric surgery (3)
- ▢ Combined oral contraceptive pills (estrogen + progestin) (4)
- ▢ Intrauterine device (IUD) (eg. Mirena or Depo Provera) (5)
- ▢ Intrauterine insemination (IUI) (6)
- ▢ In-vitro fertilization (IVF) or intracytoplasmic sperm injection (ICSI) (7)
- ▢ Laparoscopic surgery (ovarian drilling) (8)
- ▢ Metformin (insulin-sensitizing medicines) (9)
- ▢ Ovulation induction to fall pregnant (eg. Letrozole, Clomid, gonadotropins) (10)
- ▢ Never used any modern medications for PCOS (11)
- ▢ Other than above (please specify) (12) ____

Q26 **Now we would like to ask you about any traditional and complementary medicine (T&CM) that you may have used to manage PCOS.** Please tell us which of the following traditional health systems you have tried to manage PCOS?

- ▢ Acupuncture (1)
- ▢ Ayurveda (2)
- ▢ Chiropractic (3)
- ▢ Homeopathy (4)
- ▢ Naturopathy (5)
- ▢ Osteopathy (6)
- ▢ Siddha Medicines (7)
- ▢ Unani Medicines (8)
- ▢ Western herbal medicines (9)
- ▢ Traditional Chinese medicines (10)
- ▢ ⊗Never used any of the T&CM (11)
- ▢ Other than above (please specify) (12) ____

Skip To: Q27 If Now we would like to ask you about any traditional and complementary medicine (T&CM) that you may... != Ayurveda (Traditional Indian Medicine)

Skip To: End of Block If Now we would like to ask you about any traditional and complementary medicine (T&CM) that you may... = Ayurveda (Traditional Indian Medicine)

Q27 What are your reasons for not choosing Ayurvedic medicines? (multiple answers)

- ▢ Lack of scientific evidence (1)
- ▢ Poor quality of herbal medicines (2)
- ▢ Not prescribed or recommended by my doctor (3)
- ▢ Not recommended by family/friends (4)
- ▢ Not enough information (5)
- ▢ Not covered by my insurance (6)
- ▢ Not easily available (7)
- ▢ Not confident to use in conjunction with medical drugs (8)
- ▢ Takes too long to work (9)
- ▢ Smell or taste of the preparations (10)
- ▢ Not sure if it will work (11)
- ▢ ⊗Not sure/no definite reason (12)
- ▢ Other than above (please specify) (13) ____

Q28 In future, how interested you would be in trying Ayurvedic diet or medicines to manage PCOS?

- ○ Not interested at all (1)
- ○ Slightly interested (2)
- ○ Neither interested nor disinterested (3)
- ○ Very interested (4)
- ○ Extremely interested (5)

Q29 **You indicated that you have used Ayurvedic medicine (Traditional Indian Medicine) to manage PCOS, please tell us more about it.**

When did you first visit an Ayurvedic practitioner for PCOS related management?

- ○ In the last 6 months (1)
- ○ In the last 1 year (2)
- ○ In the last 1-5 years (3)
- ○ More than 5 years (4)

Q30 How long have you been using Ayurveda medicine to manage PCOS?

- ○ From past 6 months (1)
- ○ Past 1 year (2)
- ○ Past 1-5 years (3)
- ○ More than 5 years (4)

Q31 What or who most influenced your decision to use Ayurvedic medicine? (multiple answers)

- ▢ Allied health professional (eg. dietician, exercise physiologist) (1)
- ▢ Family/friends/neighbors/colleagues (2)
- ▢ Internet (3)
- ▢ Magazine or newspaper (4)
- ▢ Medical doctor/fertility specialist (5)
- ▢ Self (6)
- ▢ Television (7)
- ▢ Another complementary medicine practitioner (eg. naturopath, homeopath) (8)
- ▢ Other than above (please specify) (9) ____

Q32 What are the reasons for choosing Ayurvedic medicine? (multiple answers)

- ▢ Agree with its principles (1)
- ▢ Easily available (2)
- ▢ Cheaper/affordable (3)
- ▢ Has multiple benefits (4)
- ▢ Has natural ingredients (5)
- ▢ Helps in overall health and well-being (6)
- ▢ Lack of success with trying other treatment (7)
- ▢ Recommended by friends/family (8)
- ▢ Safe and no side effect (9)
- ▢ Traditionally accepted (10)
- ▢ Using as additional therapy alongside other treatment (11)
- ▢ Other than above (please specify) (12) ____

Q33 What forms of Ayurvedic treatment you have tried to manage the symptom of PCOS? (multiple answers)

- ▢ Ayurvedic lifestyle (eg. Ayurvedic diet/regimen) (1)
- ▢ Ayurvedic medicines for internal consumption (2)
- ▢ Ayurvedic medicines for external applications (3)
- ▢ Panchakarma (eg. Vamana, Virechana, Basti, Nasya) (4)
- ▢ Other than above (please specify) (5) ____

Q34 Have you ever had a negative reaction or unwanted side effects to Ayurvedic medicines?

- ○ Yes (1)
- ○ No (2)
- ○ Unsure (3)

Skip To: Q35 If Have you ever had a negative reaction or unwanted side effects to Ayurvedic medicines? = Yes

Skip To: Q36 If Have you ever had a negative reaction or unwanted side effects to Ayurvedic medicines? = No

Skip To: Q36 If Have you ever had a negative reaction or unwanted side effects to Ayurvedic medicines? = Unsure

---

Q35 Please write what were those negative reaction or unwanted effects.

____

____

____

____

____

Q36 On a scale of 0-10, how likely are you to recommend Ayurveda to your family members or friends to manage PCOS?

- ○ 0 (0)
- ○ 1 (1)
- ○ 2 (2)
- ○ 3 (3)
- ○ 4 (4)
- ○ 5 (5)
- ○ 6 (6)
- ○ 7 (7)
- ○ 8 (8)
- ○ 9 (9)
- ○ 10 (10)

Q37 Have you ever practised yoga since your diagnosis of PCOS?

- ○ Yes (1)
- ○ No (2)

Skip To: End of Block If Have you ever practised yoga since your diagnosis of PCOS? = Yes

Skip To: Q38 If Have you ever practised yoga since your diagnosis of PCOS? = No

Q38 What are your reasons for not choosing yoga? (multiple answers)

- ▢ Cannot find the right yoga teacher (1)
- ▢ Distance (yoga place is far from me) (2)
- ▢ Do not feel flexible enough (3)
- ▢ Do not feel fit enough (4)
- ▢ Do not feel strong enough (5)
- ▢ Financial (not enough money to pay for yoga classes) (6)
- ▢ Feel embarrassed about my body (7)
- ▢ Lack of time (to practice yoga at home or attend classes) (8)
- ▢ Lack of motivation (to do practice at home or attend classes) (9)
- ▢ Physical barriers such as injury (10)
- ▢ Other than above (please specify) (11) ____

Q39 In future, how interested you would be in practicing yoga to manage PCOS?

- Not at all interested (1)
- Slightly interested (2)
- Neither interested nor disinterested (3)
- Very interested (4)
- Extremely interested (5)

Q40 **You indicated that you have used yoga to manage PCOS, please tell us more about it.**

How long have you been practising yoga?

- ○ Last 6 months (1)
- ○ Last 1 year (2)
- ○ Last 1-5 year (3)
- ○ More than 5 years (4)

Q41 What were your specific reasons for practising yoga? (multiple answers)

- ▢ For general health and wellbeing (1)
- ▢ For emotional well-being (2)
- ▢ For stress relief (3)
- ▢ Recommended by my doctor/family physician (4)
- ▢ To manage hormonal imbalance (5)
- ▢ To maintain weight (6)
- ▢ To prevent weight gain (7)
- ▢ To reduce weight (8)
- ▢ To reduce anxiety (9)
- ▢ To reduce depression (10)
- ▢ ⊗Not sure/No definite reason (11)
- ▢ Other than above (please specify) (12)

____

Q42 Which type/style of yoga have you been practising since your diagnosis of PCOS? (multiple answers)

- ▢ Ashtanga (1)
- ▢ Bikram /Hot yoga (2)
- ▢ Iyengar (3)
- ▢ Krishnamacharya tradition or (Viniyoga) (4)
- ▢ Kundalini (5)
- ▢ Mixed style (6)
- ▢ Power Yoga (7)
- ▢ Shivananda Yoga/Yoga Vidya (8)
- ▢ Traditional Hatha Yoga (9)
- ▢ Unsure about the style (10)
- ▢ Vinyasa (11)
- ▢ ⊗Whatever is available (12)
- ▢ Other than above (please specify) (13) ____

Q43 On average, how often you currently practice yoga?

- ○ Daily (1)
- ○ 5-6 times per week (2)
- ○ 3-4 times per week (3)
- ○ 1-2 times per week (4)
- ○ Twice a month (5)
- ○ Once in a month (6)
- ○ Less than once a month (7)
- ○ I don’t practice yoga anymore (8)

Skip To: Q46 If On average, how often you currently practice yoga? = I don’t practice yoga anymore

Skip To: Q46 If On average, how often you currently practice yoga? = I don’t practice yoga anymore

Q44 Where do you practise yoga? (multiple answers)

- ▢ Home (1)
- ▢ Workplace (2)
- ▢ Yoga studio/school/institute (3)
- ▢ Fitness center/gym (4)
- ▢ Park or other public outdoor location (5)
- ▢ Other than above (please specify) (6) ____

Q45 What elements generally involves in your yoga practise? (multiple answers)

- ▢ Asana (physical postures) (1)
- ▢ Dhyana (meditation) (2)
- ▢ Mantra chanting (3)
- ▢ Pranayama (breathing practices) (4)
- ▢ Yama/Niyama (5)
- ▢ Yogic diet (6)
- ▢ Yogic kriya (eg. jala neti, vamana dhouti) (7)
- ▢ Other than above (please specify) (8) ____

Q46 What type of yoga practise would you prefer? (multiple answers)

- ▢ Individual one-on-one (1)
- ▢ Group practice (2)
- ▢ General yoga class (3)
- ▢ Yoga class designed to manage symptoms of PCOS (4)
- ▢ Other than above (please specify) (5) ____

Q47 Which of the following mode of instruction would you prefer to practise yoga? (multiple answers)

- ▢ Face to face sessions (1)
- ▢ Live online sessions (2)
- ▢ Pre-recorded online sessions (eg. internet, social media, DVD, TV) (3)
- ▢ Anything which is available/mixed (4)
- ▢ Other than above (please specify) (5) ____

Q48 Are there any challenges or barriers that prevent you from participating in yoga?

- ○ Yes (1)
- ○ No (2)
- ○ Not sure (3)

Skip To: Q49 If Are there any challenges or barriers that prevent you from participating in yoga? = Yes

Skip To: Q50 If Are there any challenges or barriers that prevent you from participating in yoga? = No

Q49 What are those challenges? (multiple answers)

- ▢ Cannot find the right yoga teacher (1)
- ▢ Distance (yoga place is far from me) (2)
- ▢ Do not feel flexible enough (3)
- ▢ Do not feel fit enough (4)
- ▢ Do not feel strong enough (5)
- ▢ Financial (not enough money to pay for yoga classes) (6)
- ▢ Feel embarrassed about my body (7)
- ▢ Lack of time (to practice yoga at home or attend classes) (8)
- ▢ Lack of motivation (to do practice at home or attend classes) (9)
- ▢ Physical barriers such as injury (10)
- ▢ Other than above (please specify) (11) ____

Q50 On a scale of 0-10, how helpful has the yoga practice been in managing PCOS?

- ○ 0 (0)
- ○ 1 (1)
- ○ 2 (2)
- ○ 3 (3)
- ○ 4 (4)
- ○ 5 (5)
- ○ 6 (6)
- ○ 7 (7)
- ○ 8 (8)
- ○ 9 (9)
- ○ 10 (10)

Q51 On a scale of 0-10, how likely are you to recommend yoga to your family members or friends to manage PCOS?

- ○ 0 (0)
- ○ 1 (1)
- ○ 2 (2)
- ○ 3 (3)
- ○ 4 (4)
- ○ 5 (5)
- ○ 6 (6)
- ○ 7 (7)
- ○ 8 (8)
- ○ 9 (9)
- ○ 10 (10)

Q52 In the past 5 years, have you tried any of the following diets to manage PCOS? (multiple answers)

- ▢ Dairy-free diet (1)
- ▢ Gluten-free diet (2)
- ▢ Higher protein diet (3)
- ▢ High omega-3 diet (4)
- ▢ Low FODMAP (Fermentable Oligosaccharides, Disaccharides, Monosaccharides, and Polyols) (5)
- ▢ Low-fat diet (6)
- ▢ Low sugar diet (7)
- ▢ Low glycemic index (low GI) or low glycemic load (low GL) diet (8)
- ▢ Mediterranean diet (9)
- ▢ Moderate carbohydrate (eg. CSIRO diet, South Beach diet) (10)
- ▢ Paleo/paleolithic diet (11)
- ▢ Reduced energy or calorie/kilojoule intake (12)
- ▢ Soy free diet (13)
- ▢ Vegan diet (14)
- ▢ Vegetarian diet (15)
- ▢ Very low carbohydrate (eg. ketogenic, Atkins) (16)
- ▢ ⊗Never tried any specific diet (17)
- ▢ Other than above (please specify) (18)

____

Q53 **Now, please lets know how active you were in the last 7 days.** How much time do you usually spend <u>sitting on a typical day?</u> (eg. sitting at work, at home, travelling in a car/bus)

- ▢ Hours per day (1-24) (1) ____
- ▢ Minutes per day (1-60) (2) ____
- ▢ ⊗Do not know/Not sure (3)

Q54 **Think about the time you spent walking in the last 7 days. (eg. walking outside or inside the home, walking from work to home)** During the last 7 days, on how many days did you <u>walk</u>?

- ○ Days per week (1-7) (1) ____
- ○ No walking (2)

Skip To: Q56 If Think about the time you spent walking in the last 7 days. (eg. walking outside or inside the hom... = No walking

Skip To: Q55 If Think about the time you spent walking in the last 7 days. (eg. walking outside or inside the hom... = Days per week (1-7)

Q55 How much time did you usually spend walking on one of those days?

- ▢ Hours per day (1-24) (1) ____
- ▢ Minutes per day (1-60) (2) ____
- ▢ ⊗Do not know / not sure (3)

Q56 **Think about all the <u>moderate activities</u> that you did in the last 7 days that cause a small increase in breathing physical activities** (eg. carrying light loads, bicycling at a regular pace, or doubles tennis etc). <u>During the last 7 days,</u> on how many days did you do these activities? Do not include walking.

- ○ Days per week (1-7) (1) ____
- ○ No moderate physical activities (2)

Skip To: Q58 If Think about all the moderate activities that you did in the last 7 days that cause a small increa... = No moderate physical activities

Skip To: Q57 If Think about all the moderate activities that you did in the last 7 days that cause a small increa... = Days per week (1-7)

Q57 How much time did you usually spend doing these activities on one of those days?

- ▢ Hours per day (1-24) (1) ____
- ▢ Minutes per day (1-60) (2) ____
- ▢ ⊗Do not know/Not sure (3)

Q58 **Now, think about all the <u>vigorous activities</u> that you did in the last 7 days such as that cause large increases in breathing or heart rate (eg. strenuous sports or recreational**

activities such as jogging, cycling, single tennis, swimming etc). During the last 7 days, on how many days did you do vigorous physical activities?

- ○ Days per week (1-7) (1) ____
- ○ No vigorous physical activities (2)

Skip To: Q60 If Now, think about all the vigorous activities that you did in the last 7 days such as that cause l... = No vigorous physical activities

Skip To: Q59 If Now, think about all the vigorous activities that you did in the last 7 days such as that cause l... = Days per week (1-7)

Q59 How much time did you usually spend doing these activities on one of those days?

- ▢ Hours per day (1-24) (1) ____
- ▢ Minutes per day (1-60) (2) ____
- ▢ ⊗Do not know / Not sure (3)

Q60 **Finally, think about the exercises that you specifically do to increase muscle strength and endurance (eg. lifting weights and push-ups)** Over the past 7 days, on how many days did you do any such exercises?

- ○ Days per week (1-7) (1) ____
- ○ No such exercises (2)

Skip To: Q61 If Finally, think about the exercises that you specifically do to increase muscle strength and endur... = Days per week (1-7)

Skip To: Q62 If Finally, think about the exercises that you specifically do to increase muscle strength and endur... = No such exercises

Q61 How much time did you usually spend doing these exercises on one of those days?

- ▢ Hours per day (1-24) (1) ____
- ▢ Minutes per day (1-60) (2) ____
- ▢ ⊗Do not know / Not sure (3)

Q62 Which of the following exercise forms has helped you to manage PCOS? (multiple answer)

- ▢ Aerobic circuit (squats, lunges, pushups, dips, torso twits) (1)
- ▢ Cardio-kickboxing (2)
- ▢ Cycling (pedal cycle outdoor) (3)
- ▢ Cycling (stationary cycle indoor) (4)
- ▢ Hight intensity interval training (HIIT) (5)
- ▢ Jumping rope (6)
- ▢ Low impact aerobic dance (7)
- ▢ Pilates/ stretching (8)
- ▢ Running or jogging (9)
- ▢ Swimming (10)
- ▢ Strength or resistance exercise (eg. weight-lifting machines, free weights, rubber bands, press-ups, squats, pushups, muscle power) (11)
- ▢ Tai-chi (12)
- ▢ Team sport (13)
- ▢ Walking (eg. brisk walking, walking a dog, leisure-time walking outside the home) (14)
- ▢ Zumba (15)
- ▢ ⊗None of the above (16)
- ▢ Other than above (please specify) (17) ____

Q63 **Finally, please tell us a few general things about yourself. Please note that this is an anonymous survey and your answers will be kept confidential.**

In which country were you born?

▼ Afghanistan (1) ... Zimbabwe (195)

Q64 In which country do you currently reside?

▼ Afghanistan (1) ... Zimbabwe (195)

Q65 How long have you been living here?

- ○ Last 1 year (1)
- ○ Last 1-5 years (2)
- ○ Last 6-10 years (3)
- ○ More than 10 years (4)

Q66 What is the highest level of school you have completed or the highest degree you have received?

- ○ No formal education (1)
- ○ Lower secondary (2)
- ○ Upper secondary (3)
- ○ Post-secondary non tertiary education (eg. vocational/ apprenticeship/ certificate) (4)
- ○ First stage of tertiary education (not leading directly to an advanced research qualification) (5)
- ○ Second stage of tertiary education (leading to an advanced research qualification) (6)
- ○ Undergraduate degree (bachelor's degree) (7)
- ○ Postgraduate (master's degree/PhD/post-doctoral degree) (8)

Q67 What is your current relationship status?

- ○ Divorced (1)
- ○ In a relationship/ de facto (2)
- ○ Married- living with a partner (3)
- ○ Single (4)
- ○ Widowed (5)
- ○ Separated (6)

Q68 Which statement best describes your current occupation? (multiple answer)

- ▢ Employed full time (1)
- ▢ Employed part-time (2)
- ▢ Home duties/ caring for family or children (3)
- ▢ Retired (4)
- ▢ Self-employed/Freelance (5)
- ▢ Studying (eg. going to school/college/university) (6)
- ▢ Unemployed- Looking for work (7)
- ▢ Unemployed- not looking for work (8)
- ▢ Unable to work because of symptoms/treatment of PCOS (9)
- ▢ Volunteer work (10)
- ▢ Other (please specify) (11) ____

Q69 Have you ever been pregnant?

- ○ Yes (1)
- ○ No (2)

Skip To: End of Block If Have you ever been pregnant? = No

Q70 Have you ever needed any treatments to help you become pregnant?

- ○ Yes (1)
- ○ No (2)

Q71 How many biological children do you have?

- ▢ None (1)
- ▢ Currently Pregnant (2)
- ▢ 1 (3)
- ▢ 2 (4)
- ▢ More than 2 (5)

Q72 Finally, is there anything else you would like to share about your experience of PCOS?

____
